# Maternal-Infant Correlation of Multidrug-Resistant *Staphylococcus aureus* Carriage: A Prospective Cohort Study

**DOI:** 10.3389/fped.2018.00384

**Published:** 2018-12-05

**Authors:** Jialing Lin, Zhenjiang Yao

**Affiliations:** Department of Epidemiology and Health Statistics, School of Public Health, Guangdong Pharmaceutical University, Guangzhou, China

**Keywords:** *Staphylococcus aureus*, multidrug resistant, mothers, infants, cohort

## Abstract

**Objectives:** We aim to assess the correlation of multidrug-resistant *Staphylococcus aureus* (MDR *S. aureus*) carriage between mothers and their newborn infants.

**Materials and Methods:** We conducted a prospective cohort study of mothers and their newborn infants in two hospitals in Shenzhen, China, from August to November 2015. We collected demographic and clinical information from mothers and newborn infants by face-to-face questionnaires and medical datasets. Serial swabs were collected from mothers and their newborn infants for further experiments. Maternal-infant correlation was assessed using the Poisson regression model.

**Results:** The prevalence of MDR *S. aureus* vaginal carriage in mothers was 4.7% (86/1834). The incidence of MDR *S. aureus* carriage in newborn infants was 1.3% (23/1834). The adjusted relative risk and 95% confidence interval of maternal-infant MDR *S. aureus* carriage was 7.63 (2.99–19.49). Six MDR *S. aureus* maternal-infant pairs were concordant. The phenotypic and molecular characteristics of MDR *S. aureus* isolates were similar between mothers and their newborn infants.

**Conclusion:** MDR *S. aureus* vaginal carriage in mothers was associated with an increased risk for MDR *S. aureus* carriage in their newborn infants.

## Introduction

*Staphylococcus aureus* (*S. aureus*) is one of the most important human bacterial pathogens that cause nosocomial and community infections, and results in substantial morbidity and mortality (1). Multidrug-resistant *S. aureus* (MDR *S. aureus*) is of greater significance because of its more severe clinical outcomes (2).

Those persons who are colonized with MDR *S. aureus* have a higher risk for subsequent infections than non-colonized individuals (3, 4). Most infections are caused by the same MDR *S. aureus* strain that previously colonized the person (5). In response to these findings, there has been increasing attention to the detection of MDR *S. aureus* carriage, with subsequent decolonization of carriers as a potential method for the prevention of MDR *S. aureus* infection.

Most of the available data on the detection of MDR *S. aureus* carriage have been in hospitalized non-pregnant adults (6, 7). Despite the fact that MDR *S. aureus* outbreaks in infants have been linked to the infected or colonized mother, there are limited data on MDR *S. aureus* carriage rates among mothers or on the risk of transmission of MDR *S. aureus* from pregnant MDR *S. aureus* carriers to their newborn infants (8, 9). The maternal-infant relatedness of MDR *S. aureus* carriage is of particular interest.

Accordingly, we determined whether vaginal carriage of MDR *S. aureus* in the mothers is independently correlated with MDR *S. aureus* carriage in their newborn infants. Antimicrobial susceptibility, virulence factors, and clonality of isolates were also evaluated. The hypothesis of this study is that there is maternal-infant vertical MDR *S. aureus* carriage.

## Materials and Methods

This study was approved by the Ethics Committee of Guangdong Pharmaceutical University and was performed in accordance with the approved guidelines. Written informed consent was obtained from the mothers and their newborn infants involved in the study before enrollment.

This prospective cohort study was conducted in two hospitals in Shenzhen, China, between August and November 2015. The two hospitals, Longhua Central Hospital and Guanlan People's Hospital, are large hospitals whose obstetric services deliver between 10,000 and 15,000 neonates per year; these are two of the largest delivery hospitals in China. The target populations were mothers and their newborn infants. Chinese mothers with gestation between 35 and 40 weeks were voluntarily included. Mothers with twin or multiple gestations, cesarean section, or acute diseases were excluded. Newborn infants without sample collection were also excluded. The sample size was calculated using the power two proportions method with 4.00% proportion (approximate prevalence of *S. aureus* in the newborns), 4.00 ratio, 0.05 alpha, and 0.80 power. Taking a 10% loss of follow-up into consideration, the estimated minimum sample size of this study was 107 for each group.

A face-to-face questionnaire was administered by trained personnel that aimed to collect demographic information and information regarding potential factors influencing MDR *S. aureus* carriage during pregnancy. Medical records of the mothers and their newborn infants were reviewed by two members of the study team who were blinded to the maternal and infant MDR *S. aureus* carriage status.

Sterile swabs moistened with sterile saline water were used to get specimens from the vagina of the enrolled mothers prior to delivery by trained personnel. Infant specimens for MDR *S. aureus* were sampled from the nasal cavity, ear canal, oral cavity, and umbilicus by trained nurses immediately after birth. All specimens were then inoculated into enrichment broth tubes containing 1% tryptone, 7.5% sodium chloride, 1% mannitol, and 0.25% yeast extract.

All included mothers were informed of the research question and outcome measures by well-trained staff in person before participating. The trained personnel informed the mothers of the results of *S. aureus* carriage by telephone calls. Chinese mothers with gestation between 35 and 40 weeks were voluntarily included in this study. Included mothers were asked to complete face-to-face questionnaires and collected vaginal samples by trained personnel.

Swabs were inoculated into mannitol salt agar plates at 37 ± 1°C for 24 h of incubation. Isolates were confirmed to be *S. aureus* by a combination of gram staining, catalase reaction, hemolysis test, DNase test, and coagulase tests.

Antimicrobial susceptibility testing was performed by the Kirby-Bauer disk diffusion method according to the Clinical and Laboratory Standards Institute guidelines of 2015. The following antimicrobial disks were used: cefoxitin, clindamycin, rifampicin, moxifloxacin, tobramycin, gentamicin, sulfamethoxazole-trimethoprim, linezolid, and erythromycin. Isolates were defined as resistant if they were resistant or intermediate to the antimicrobial disk. *S. aureus* isolates were classified as MDR if they were resistant to no less than three antibiotic classes (10).

Multilocus sequence typing (MLST) was used to type MDR *S. aureus*, which involved sequencing the seven housekeeping genes (11). Then, the sequence types (STs) and allelic profiles were confirmed by querying the MLST database (http://eburst.mlst.net). STs were clustered into a clonal complex (CC) by using the eBURST software program (Department of Infectious Disease Epidemiology, Imperial College London, London, UK; http://eburst.mlst.net). The presence of virulence genes including Panton-valentine leukocidin (*Pvl*), toxic shock syndrome toxin (*Tst*), exfoliative toxin A (*Eta*), and exfoliative toxin B (*Etb*) using polymerase chain reaction assays as in previous studies was determined as previously described (12, 13).

All data were entered in duplicate into the EpiData version 3.0 database (The EpiData Association, Odense Denmark). Missing data were excluded. Categorical variables were compared by Pearson's chi-squared test or the Fisher exact test when appropriate. To identify variables that might confound the correlation of MDR *S. aureus* carriage between mothers and newborn infants, influencing factors with a *P*-value < 0.2 were identified as adjustment variables using the Poisson regression model to estimate the correlations of isolates between mothers and newborn infants. Relative risks (RRs) and 95%confidence intervals (CIs) were used to assess the maternal-infant relatedness of MDR *S. aureus* carriage. The relationship between populations and main STs of MDR *S. aureus* isolates was illustrated by a minimum spanning tree (PHYLOVIZ software version 2.0; http://www.phyloviz.net). A two-sided *P*-value < 0.05 was considered as being of statistical significance. All analyses were performed using Stata version 14.2 (College Station, Texas, USA).

## Results

Overall, 1968 mothers were preliminarily enrolled, but only 1,834 mothers and their newborn infants met the inclusion criteria and consented to participate in this study. We found that there were 133 *S. aureus* isolates and the prevalence of MDR *S. aureus* vaginal carriage in mothers was 4.7% (86/1834). There was no statistical difference of MDR *S. aureus* carriage in mothers from the two different hospitals (χ^2^ = 0.029, *P*-value = 0.865).

There were 60 *S. aureus* isolates and the incidence of MDR *S. aureus* carriage in newborn infants was 1.3% (23/1834). There was no statistical difference of MDR *S. aureus* carriage in newborn infants from the two different hospitals (χ^2^ = 0.846, *P*-value = 0.358).

MDR *S. aureus* vaginal carriage in mothers (χ^2^ = 69.163, *P*-value < 0.001) was significantly correlated with MDR *S. aureus* carriage in their newborn infants. However, there were no other significant variables correlated with MDR *S. aureus* carriage in infants. More details can be found in Table 1.

**Table 1:** Characteristics of multidrug-resistant *Staphylococcus aureus* carriage among newborn infants in Shenzhen, 2015 [n (%)].

**Characteristics**	**Non-MDR *S. aureus***	**MDR *S. aureus***	**χ^2^**	***P*-value**
	**(*n* = 1811)**	**(*n* = 23)**		
**MOTHERS**				
Vaginal carriage	80 (4.4)	6 (26.1)	23.862	< 0.001
Age, year (>35)	137 (7.6)	2 (8.7)	NA	0.692^a^
Education (below high school)	1280 (70.7)	20 (87.0)	2.916	0.088
Average monthly income, yuan (< 5000)	1013 (55.9)	17 (73.9)	2.981	0.084
Natural impregnation	1786 (98.6)	23 (100.0)	NA	1.000^a^
First pregnancy	602 (33.2)	5 (21.7)	1.357	0.244
First parturition	916 (50.6)	9 (39.1)	1.191	0.275
History of abortion	751 (41.5)	10 (43.5)	0.038	0.846
Frequency of vaginal examination after hospitalization ( ≤ 2)	947 (52.3)	13 (56.5)	2.108	0.349
Vaginitis	157 (8.7)	1 (4.4)	NA	0.715^a^
Premature rupture of membranes	8 (0.4)	0 (0.0)	NA	1.000^a^
Days of hospitalization (>3)	1380 (76.2)	16 (69.6)	0.550	0.458
Weeks of pregnancy (< 37)	75 (4.1)	1 (4.4)	NA	1.000^a^
Tobacco use during pregnancy	5 (0.3)	0 (0.0)	NA	1.000^a^
Antibiotic use during pregnancy	120 (6.6)	1 (4.4)	NA	1.000^a^
Mammal pet owner	71 (3.9)	2 (8.7)	NA	0.232^a^
**NEWBORN INFANTS**				
Male gender	977 (54.0)	14 (60.9)	0.438	0.508
Birth weight, grams (< 2500)	133 (7.3)	3 (13.0)	NA	0.240^a^
Admission to neonatology ward	158 (8.7)	1 (4.4)	NA	0.715^a^
Apgar 1st min ≤ 3	4 (0.2)	0 (0.0)	NA	NA^b^
Apgar 5th min ≤ 6	4 (0.2)	0 (0.0)	NA	NA^b^

*MDR S. aureus, multidrug-resistant Staphylococcus aureus; n, number of isolates; NA, not applicable*.

a *The P-values were calculated with the Fisher's exact test*.

b *No estimate of the P-value is provided owing to the lack of occurrence of the outcome of interest in at least one group*.

After adjusting for the age of mothers, education level of mothers, average monthly income, and gender of newborn infants, MDR *S. aureus* vaginal carriage in mothers was still a risk factor for MDR *S. aureus* carriage in newborn infants [adjusted RR (aRR) = 7.63; 95%CI, 2.99–19.49; *P*-value < 0.001]. More details can be found in Table 2.

**Table 2:** Correlations of multidrug-resistant *Staphylococcus aureus* carriage between mothers and their newborn infants in Shenzhen, 2015.

**RR category**	**RR/aRR (95% CI)**	**z**	***P*-value**
Crude RR	7.17 (2.83–18.19)	4.15	<0.001
Adjusted RR^a^	7.63 (2.99–19.49)	4.24	<0.001

*MDR S. aureus, multidrug-resistant Staphylococcus aureus; RR, relative risk; aRR, adjusted relative risk; CI, confidence interval*.

a *It was adjusted for age of mothers, education level of mothers, average monthly income, and gender of newborn infants*.

The most predominant proportion of antibiotic resistance in the 86 MDR *S. aureus* isolates from mothers was penicillin (97.7%), followed by erythromycin (73.3%), clindamycin (64.0%), tobramycin (38.4%), cefoxitin (26.7%), trimethoprim-sulfamethoxazole (20.9%), moxifloxacin (19.8%), gentamicin (12.8%), linezolid (5.8%), and rifampicin (2.3%). The most predominant proportion of antibiotic resistance in 23 MDR *S. aureus* isolates from the newborn infants was penicillin (95.7%), followed by erythromycin (91.3%), clindamycin (69.6%), cefoxitin (47.8%), trimethoprim-sulfamethoxazole (30.4%), gentamicin (21.7%), tobramycin (21.7%), rifampicin (21.7%), moxifloxacin (13.0%), and linezolid (8.7%). The proportion of resistant rifampicin (*P*-value = 0.004) in MDR *S. aureus* isolates was significantly different between mothers and their newborn infants. More details can be found in Table 3.

**Table 3:** Phenotypic and genetic characteristics of multidrug-resistant *Staphylococcus aureus* isolates between mothers and their newborn infants in Shenzhen, 2015 [n (%)].

**Characteristics**	**Mothers**	**Infants**	**χ^2^**	***P*-value**
	**(*n* = 86)**	**(*n* = 23)**		
**RESISTANCE PHENOTYPE (RESISTANT)**				
Cefoxitin	23 (26.7)	11 (47.8)	3.758	0.053
Erythromycin	63 (73.3)	21 (91.3)	3.344	0.067
Penicillin	84 (97.7)	22 (95.7)	NA	0.513^a^
Gentamicin	11 (12.8)	5 (21.7)	NA	0.322^a^
Clindamycin	55 (64.0)	16 (69.6)	0.252	0.616
Rifampicin	2 (2.3)	5 (21.7)	NA	0.004^a^
Linezolid	5 (5.8)	2 (8.7)	NA	0.637^a^
Moxifloxacin	17 (19.8)	3 (13.0)	NA	0.558^a^
Trimethoprim-sulfamethoxazole	18 (20.9)	7 (30.4)	0.928	0.336^a^
Tobramycin	33 (38.4)	5 (21.7)	2.211	0.137
**VIRULENCE GENE (POSITIVE)**				
*Pvl*	3 (3.5)	1 (4.4)	NA	1.000^a^
*Tst*	2 (2.3)	0 (0.0)	NA	1.000^a^
*Eta*	1 (1.2)	2 (8.7)	NA	0.112^a^
*Etb*	0 (0.0)	0 (0.0)	NA	NA^b^

*MDR S. aureus, multidrug-resistant Staphylococcus aureus; n, number of isolates; Pvl, panton-valentine leukocidin; Tst, toxic shock syndrome toxin; Eta, exfoliative toxin A; Etb, exfoliative toxin B; NA, not applicable*.

a *The P-values were calculated with the Fisher's exact test*.

b *No estimate of the P-value is provided owing to the lack of occurrence of the outcome of interest in at least one group*.

The results indicated that the proportions of virulence genes of isolates in both mothers and their newborns were low. The proportion of the positive *Pvl* gene in MDR *S. aureus* isolates was significantly different between mothers and newborn infants (Fisher's exact test, *P*-value = 0.041). There were no significant differences of virulence genes in MDR *S. aureus* isolates between mothers and their newborn infants. More details can be found in Table 3.

The most common CC of 86 MDR *S. aureus* isolates in mothers was CC5 (51.2%), followed by CC7 (19.8%), CC59 (14.0%), CC88 (5.8%), CC398 (3.5%), CC20 (2.3%), CC45 (2.3%), and CC121 (1.2%). The most common CC of 23 MDR *S. aureus* isolates in newborn infants was CC5 (38.1%), followed by CC59 (28.6%), CC45 (9.5%), CC88 (9.5%), CC7 (4.8%), CC22 (4.8%), and CC398 (4.8%).

**Figure 1:**
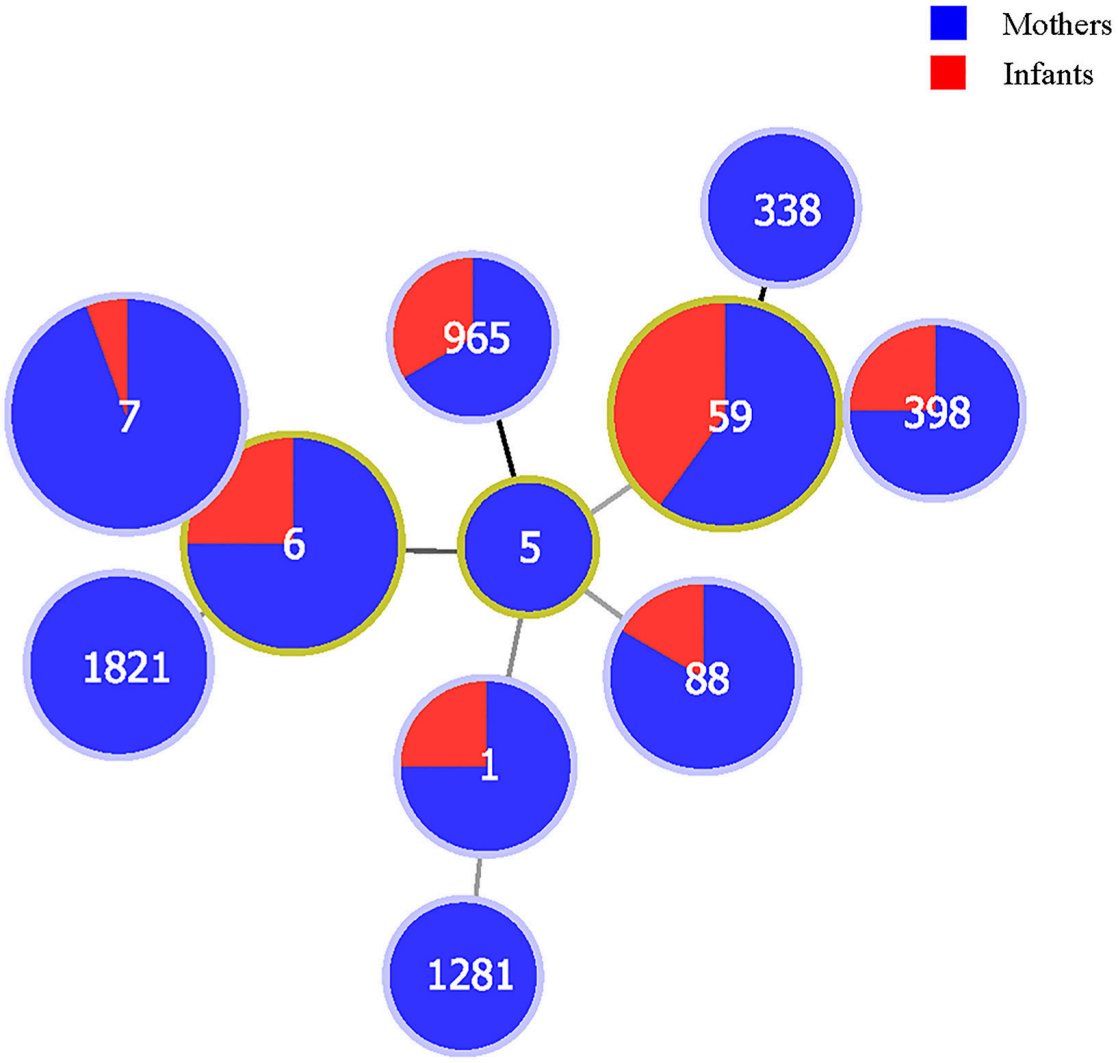
The correlations between populations (mothers and newborn infants) and sequence types of multidrug-resistant *Staphylococcus aureus* isolates in Shenzhen, 2015. The numbers in the circles are the sequence types. The size of the circles represents the number of isolates. The distance of lines between each circle represents the degree of the relationship.

The minimum spanning tree demonstrated a good concordance of certain STs between mothers and their newborn infants; for example, MDR *S. aureus* isolates belonging to ST1, ST6, ST7, ST59, ST88, ST398, and ST965 were found in both mothers and newborn infants. More details can be found in Figure 1.

Overall, there were six MDR *S. aureus* maternal-infant pairs. These six MDR *S. aureus* maternal-infant pairs were concordant, with the same phenotypic and molecular characteristics. More details can be found in Table 4.

**Table 4:** Characteristics of six maternal-infant pairs with multidrug-resistant *Staphylococcus aureus* isolates in Shenzhen, 2015.

**Pair**	**Population**	**CC**	**ST**	**Antibiotic resistance**										**Virulence**			

				**FOX**	**EM**	**PCN**	**GM**	**CM**	**REP**	**LZD**	**MXF**	**TMP/SMX**	**TOB**	***Pvl***	***Tst***	***Eta***	***Etb***
1	Mother	5	1	–	+	+	+	–	–	–	–	+	–	–	–	–	–
	Infant	5	1	–	+	+	+	–	–	–	–	+	–	–	–	–	–
2	Mother	5	6	–	+	+	–	+	–	–	–	–	–	–	–	–	–
	Infant	5	6	–	+	+	–	+	–	–	–	–	–	–	–	–	–
3	Mother	5	188	+	+	+	–	+	–	–	+	+	–	–	–	–	–
	Infant	5	188	+	+	+	–	+	–	–	+	+	–	–	–	–	–
4	Mother	5	965	+	+	+	–	+	–	–	–	–	–	–	–	–	–
	Infant	5	965	+	+	+	–	+	–	–	–	–	–	–	–	–	–
5	Mother	59	59	+	+	+	–	+	–	–	–	–	+	–	–	–	–
	Infant	59	59	+	+	+	–	+	–	–	–	–	+	–	–	–	–
6	Mother	59	59	+	+	+	–	–	–	–	–	–	–	–	–	–	–
	Infant	59	59	+	+	+	–	–	–	–	–	–	–	–	–	–	–

*MDR S. aureus, multidrug-resistant Staphylococcus aureus; CC, clonal complex; ST, sequence type; FOX, cefoxitin; EM, erythromycin; PCN, penicillin; GM, gentamicin; CM, clindamycin; RFP, rifampicin; LZD, linezolid; MXF, moxifloxacin; TOB, tobramycin; TMP/SMX, trimethoprim-sulfamethoxazole; MDR, multidrug-resistance, resistant to no less than three antibiotic classes; Pvl, panton-valentine leukocidin; Tst, toxic shock syndrome toxin; Eta, exfoliative toxin A; Etb, exfoliative toxin B; +, positive; −, negative*.

## Discussion

Methicillin-Resistant *Staphylococcus aureus* (MRSA) carriage between mothers and their infants has been reported, but no previous studies reported the proportions of MDR *S. aureus* in both mothers and infants. The prevalence of MRSA in MDR *S. aureus* among mothers and their newborns was 26.7 and 47.8%, respectively. On the contrary, the prevalence of MDR *S. aureus* in MRSA among mothers and their newborns were 88.5 and 91.7%, respectively. These results were similar to current observed studies that MRSA is always MDR *S. aureus*, but MDR *S. aureus* is not necessarily MRSA (7, 11). Therefore, it is significant to assess the mother-infant relationship of MDR *S. aureus*. An American study reported that the proportion of MDR *S. aureus* carriage in community residents was 6.9% (14). Other previous studies have reported that the prevalence of *S. aureus* vaginal carriage in mothers (pregnant women) and infants ranged from 0.96–12.6% (15–18) to 5.4–17.7% (15, 16, 18–21), respectively. Therefore, the results suggested that MDR *S. aureus* carriage in our target populations were moderate when compared with other countries and regions.

This prospective cohort study identified risk factors for MDR *S. aureus* carriage in newborn infants and assessed the risk of MDR *S. aureus* vaginal carriage of mothers for MDR *S. aureus* carriage in their newborn infants. The gender of the newborn infants was not associated with MDR *S. aureus* isolates in this study, which was different to previous studies (22, 23). The mechanism between gender and MDR *S. aureus* carriage requires further exploration. No previous studies have particularly explored the maternal-infant relatedness of MDR *S. aureus* carriage. Therefore, we compared our results with other previous maternal-infant *Staphylococcus aureus* carriage. The aRR of maternal-infant MDR *S. aureus* carriage in our study was 7.63. We found that it was much higher than other previous studies, which reported that the RRs of maternal-infant *Staphylococcus aureus* carriage ranged from 2.04 to 5.70 (21, 24–26). The results demonstrated that maternal-infant MDR *S. aureus* transmission is much more hazardous.

There were few significant differences on phenotypic and virulence genetic characterizations in MDR *S. aureus* isolates between mothers and their newborn infants. We observed good consistency on certain STs of MDR *S. aureus* isolates between mothers and their newborn infants, such as ST1, ST6, ST7, ST59, ST88, ST398, and ST965. These STs were also reported in other previous studies (20, 27). Furthermore, we found that six concordant maternal-infant MDR *S. aureus* pairs had the same phenotypic and molecular characteristics. These results could further verify the homology of maternal-infant MDR *S. aureus* carriage.

In the current study, we found that more than 5% of MDR *S. aureus* isolates were resistant to linezolid and the rate was higher in isolates from newborns. This was not usual, and it could raise major issues in the case of infection with these strains (28). Healthcare workers should pay greater attention to mothers and newborns with linezolid resistance.

This study has some limitations. First, the correlation of MDR *S. aureus* carriage between mothers and infants needs to be explored further because of the limited sample size in this study. Second, whole-genome sequencing would further strengthen this study. Third, the potential importance of environmental contamination in MDR *S. aureus* carriage would need to be further explored. Fourth, participants in this study were volunteers, recruited using a convenient sampling approach. It is possible that selection bias may have occurred. Last, we did not follow up with the newborns because of insufficient financial and human support.

In summary, we identified vertical maternal-infant transmission of MDR *S. aureus* carriage in newborn infants. Newborn infants born with maternal MDR *S. aureus* carriage appear to be at higher risk of MDR *S. aureus* carriage. Accordingly, routine surveillance for MDR *S. aureus* carriage in mothers may be indicated. Prevention measures focused on controlling the spread of MDR *S. aureus* in mothers could be a more effective strategy when outbreaks of MDR *S. aureus* in newborn infants occur. Further work should seek to elucidate the potential role of maternally derived antibodies in modifying MDR *S. aureus* carriage risk in newborn infants.

## Author Contributions

ZY designed the study, performed the data analysis, and revised the manuscript. JL collected the information, conducted the experiments, performed the data analysis, and wrote the manuscript.

### Conflict of Interest Statement

The authors declare that the research was conducted in the absence of any commercial or financial relationships that could be construed as a potential conflict of interest.
